# The embryo as moral work object: PGD/IVF staff views and experiences

**DOI:** 10.1111/j.1467-9566.2008.01083.x

**Published:** 2008-07

**Authors:** Kathryn Ehrich, Clare Williams, Bobbie Farsides

**Affiliations:** 1 Centre for Biomedicine and Society, King's CollegeLondon; 2 Brighton and Sussex Medical School

**Keywords:** work object, embryo, PGD, ethnography, ethics

## Abstract

We report on one aspect of a study that explored the views and experiences of practitioners and scientists on social, ethical and clinical dilemmas encountered when working in the field of preimplantation genetic diagnosis (PGD) for serious genetic disorders. The study produced an ethnography based on observation, interviews and ethics discussion groups with staff from two PGD/IVF Units in the UK. We focus here on staff perceptions of work with embryos that entails disposing of ‘affected’ or ‘spare’ embryos or using them for research. A variety of views were expressed on the ‘embryo question’ in contrast to polarised media debates. We argue that the prevailing policy acceptance of destroying affected embryos, and allowing research on embryos up to 14 days leaves some staff with rarely reported, ambivalent feelings. Staff views are under-researched in this area and we focus on how they may reconcile their personal moral views with the ethical framework in their field. Staff construct embryos in a variety of ways as ‘moral work objects’. This allows them to shift attention between micro-level and overarching institutional work goals, building on Casper's concept of ‘work objects’ and focusing on negotiation of the social order in a morally contested field.

## Introduction

The field of preimplantation genetic diagnosis (PGD) is a morally contentious one, including such issues as when life begins and when it begins to be morally significant; the notion of a ‘pre-embryo’; the selection of some embryos and the discarding of others; and when it can be justified to use embryos for research (*e.g.* Spallone 1989, Farsides *et al.* 2005, Krones *et al.* 2006). In the UK, media debates about the ‘embryo question’ have tended to be polarised, but policy and professional commitments to offering patients a ‘choice’ of embryos have coalesced in an ‘official’ account of the ethics of PGD, including acceptance of the destruction of ‘affected’ or ‘spare’ embryos and of a 14-day limit for embryo research. The expression in legislation and regulation of this account might be assumed to some extent both to reflect and to underpin professional attitudes towards the status and use of embryos. However, one effect of the achievement of a workable official framework is that further public debate on the ‘embryo question’ in particular has been discouraged, and little has been reported of the experiences and views of the practitioners and scientists who carry out this novel form of genetic and reproductive technology. In particular, little is known of how staff understand the moral meaning and significance of embryos, and how this relates to work goals in the application of new reproductive technologies.

### Policy background

As Mary Warnock (2006) has recently commented, ‘the relation between private morality and a public policy that is democratically acceptable is a subtle and complicated interrelation’. New genetic technologies intensify long-standing contrasts between the public and private spheres (Parker 2000: 160). However, it is rarely acknowledged that professionals working in this area need to straddle a number of ethical boundaries such as accommodating differences between private and public views and interests, including their own (Farsides *et al.* 2005, Ehrich *et al.* 2006, Wainwright *et al.* 2006).

In the UK, the Human Fertilisation and Embryology Authority (HFEA), established by the *HFE Act 1990*, is responsible for licensing all forms of assisted reproduction and consequently PGD, which uses IVF technology in order to create and access the three-day-old embryo for biopsy. The establishment of the HFEA was crucial to the passage of the Act, because it represented the means by which scientific and medical research would be subject to public acceptance and accountability, and as such has been argued to be a form of ‘sociological’ regulation (Franklin and Roberts 2006). Another principle on which the HFEA's work rests is acceptance of the pre-14-day embryo as lacking distinct individual moral status. This paved the way for PGD and research on embryos, but was only reached after nearly a decade of political turmoil stemming, on the one hand, from long-standing conflicts over the status of embryos in relation to abortion; and, on the other, claims about therapeutic benefits made by the medical and scientific communities and affected families. In the last stages of debate prior to the 1990 Act being passed, an emphasis on the benefits of testing embryos for serious genetic conditions was used to garner and galvanise support for research on embryos (Mulkay 1997). As Warnock recounts, ‘Utilitarian considerations came in, insofar as the benefits to the infertile and to society at large were cited in justification of research. The consequences to the embryos were discounted’ (Warnock 2006).

In subsequent legislation, and guidance from the HFEA since 1990, references to the destruction of embryos have been confined to issues of storage and the obligation of licensees to allow embryos to perish unless they hold the required consents from the relevant parties (*e.g. HFE Act 1990*, c.37, section 14). In 2001, the Human Fertilisation and Embryology (Research Purposes) Regulations Act also conveyed the impression that pre-14-day-old embryos, though worthy of ‘respect’, lacked sufficient moral status to outweigh the presumed common good of finding cures for serious disease (Deckers 2005). In the same year, the European Society of Human Reproduction and Embryology Task Force on Ethics and Law (2001) also concluded: ‘the cryopreserved pre-implantation embryo is not *a full person*, and considering the pre-implantation embryo as a step in the achievement of a parental project, we do not object to the disposal of the pre-implantation embryo … or to the donation for research’ (2001: 1047, italics added).

More recently, the *HFE Act 1990* has been reviewed, although the consultation document states that ‘the Government … does not intend to open up the most fundamental aspects of the Act, or those aspects that have been extensively and conclusively debated in recent years’, for example acceptance of the creation and use of embryos for research, and the associated destruction of embryos (Department of Health 2005: 63).

In short, the question of *destruction* of embryos as a consequence of fertility treatment, PGD, and research on embryos up to 14 days old, appears to have been largely laid to rest as a moral, clinical or political issue, despite the efforts of many interested parties (especially pro-life and religious groups) to keep it on the agenda. Further public debate on this specific issue does not appear to be encouraged, although consultations seeking professional and public views on other ethical issues continue as part of the ongoing work of the HFEA and of the current review of the *HFE Act 1990*.

### A polarised media debate

Despite this avoidance of further debate on destruction of embryos, the utility of affected or non-viable PGD and in-vitro fertilisation (IVF) embryos for stem cell research (as one of the more controversial beneficiaries of PGD and IVF embryos not used for treatment), has reopened some of the previous discussions (Franklin 1997, Parry 2003). Williams, Kitzinger and Henderson (2003) examine discourses surrounding the embryo in mass media discussions of stem cell research and note that the mass media structure these discourses as a binary debate between those who regard such research as an abuse of embryos, and those who regard the benefits as outweighing any risks or ethical dilemmas (Parry 2003: 797). Opponents of stem cell research, echoing earlier opposition to IVF and PGD, focus on when life is morally significant, and how death is defined, speaking of embryos being ‘killed’, in language rigorously avoided by proponents on the other side of the debate (2003: 803). While it is important to note that media and policy discourse take place in different arenas, Parry (2003) argues that the policymaking process relating to the regulation of stem cell research has followed this pattern of polarisation, especially around the ‘embryo question’, and that these debates have reinvigorated the ‘pro-life’ campaign against the use of embryos.

The polarising of debates in the media about the embryo may reflect contrasting views in a broader audience, but many professionals in this field feel misrepresented in that arena. Similarly, it is not clear that the trend in policy making which seems to favour the utilitarian view of embryos is a complete and accurate picture of the views of those working in the field. We argue that there is a need to investigate clinical and scientific professionals’ views as there is some evidence (Kerr, Cunningham-Burley and Tutton 2007, Kerr and Franklin 2006) that contrary to possible assumptions about professional views, they may include a wide range of perspectives, including feelings of uncertainty and ambivalence.

### Professional ambivalence

We now turn to a discussion of ambivalence of relevance to this official account and to our investigation of the views and experiences of those working in this field.

Bauman (1989, 1991) claims that the modernist impulse towards social control and order is inevitably accompanied by a counter effect of ambiguity and uncertainty. He argues that in ‘liquid’ or late modernity, these feelings of uncertainty and ambivalence are increasingly privatised, thwarting their radical collective power. Rather than suppressing the ‘messiness’ of ambivalence and uncertainty, we should embrace it as an act of moral resistance to rationalising forces. Another consequence of modernity (Giddens 1990) is that institutions engaging in the production of various kinds of social order may engender similar counter effects (mistrust, challenges to forms of knowledge) and become caught up in ‘the permanent monitoring of social practices in the light of incoming information about these practices themselves’ (Giddens 1990: 38). ‘Institutional reflexivity’ describes the capacity for perpetual monitoring of social practices which are constantly examined, reframed and reflexively managed. Management of dissenting views, perhaps including the personal management of ambivalence, becomes part of a successful reflexive response in conditions of modernity and counter-modernity (Kerr and Cunningham-Burley 2000, Kerr, Cunningham-Burley and Tutton 2007).

Kerr and Cunningham-Burley argue that reflexive modernity, characterised by ‘tensions between reflexivity and objectivity; doubt and certainty; choice and coercion; and change and convention’ (2000: 285), is more manifest in lay responses to science, technology and institutions of the new genetics than it is a feature of professional rhetoric in this field. They contrast lay ambivalence about the new human genetics with the professional project of expanding ‘ethically acceptable’ clinical genetic services (2000: 295), and argue that the little dissent that does exist is carefully managed. ‘Institutional reflexivity’, through professional influence on the media and government, co-option of (for example) bioethicists, and domination of public debate, sets

clear boundaries around which areas are not open for social consideration, [and] also allows professionals, associated with the new human genetics, to manage concern without undermining research and practice … By concentrating on knowable and confinable risks associated with the *application* of genetic technologies, professionals also side-step more fundamental questions about the values embedded in the new human genetics … The new human genetics and eugenics are demarcated on the basis of their practitioners’ goals and motives (as opposed to the consequences of their work) (Kerr and Cunningham-Burley 2000: 294–295 italics in original).

While such side-stepping may have served to fend off public criticism of their work, we are concerned in this paper with the silencing effect such boundary-making may have on healthcare professionals themselves within their own spheres of practice. We did not find evidence of what Bauman (1989) has described as a bureaucratic and functional division of labour that separates efficiency and technical performance of tasks off from their moral content; rather, we encountered a range of views and processes through which some staff were confronted with the need to negotiate their relationship to the ethical framework in which they work, including in particular the suppression of further debate on the status of the embryo.

As Kerr (2004) states, there is a relative lack of literature on professional practice in the area of human genetics, and, ‘a tendency to black box professionals’ roles, alongside the genes, knowledge and technologies with which they work’ (2004: 163), resulting in a lack of knowledge about professional uncertainty and ambivalence. More recently, some evidence has emerged that dissent from the official account in this morally contested field might be difficult to express in a workplace context (Kerr, Cunningham-Burley and Tutton 2007, Kerr and Franklin 2006). We agree that: ‘The challenge to social scientists is to open up spaces for ambivalence to be more actively and self-consciously articulated and explored, by both professionals and lay people as well as the many hybrids between them (Kerr, Cunningham-Burley and Tutton 2007: 68).

In this paper we contribute to this small but growing body of literature by exploring some ambivalent and/or diverse views amongst a multidisciplinary group of PGD/IVF staff, and investigate how personal views which diverge from ‘official’ constructions of the embryo may be managed in relation to the collective enterprise in which staff are engaged. Our findings support a strand of Kerr and Cunningham-Burley's (2000) argument that focusing on work goals rather than fundamental questions of value may be an important strategy that allows the work to continue, but we provide data on a specific topic area that is not addressed in Kerr and Cunningham-Burley's (2000) and Kerr, Cunningham-Burley and Tutton's (2007) reports of ambivalence in this field: professional views on the destruction of embryos as part of IVF and PGD.

We would like to point out that in this paper, we acknowledge that individual members of staff may need to engage in internal processing of feelings that arise regarding the ethical issues surrounding destruction of embryos as part of their work, but our main focus is on the negotiation of such feelings in the context of a particular regulatory and professional environment. Thus, we address the phenomenon that, from a variety of personal positions, staff can be seen as acting reflexively not so much in the sense of managing *public* ambivalence, as Kerr and Cunningham-Burley (2000) argue, nor entirely as the subjects of external ethical management, but rather they engage at this level in a reflexive process of creating a workable ethical practice between the personal, professional and regulatory spheres. In a further reflexive turn, this can be valued positively as it can be argued that this level of ethical consciousness enhances their work and the service they provide to their clients (Farsides, Williams and Alderson 2005, Franklin and Roberts 2006, Kerr, Cunningham-Burley and Tutton 2007).

### The embryo as ‘moral work object’

We start from the recognition that embryos are socially, culturally and politically constructed (Williams *et al.* 2003), and we consider how different constructions of the embryo can exist within the negotiated order of ethical principles and practice in one assisted conception unit. Embryos have been thought of as ‘social objects’, *i.e.* objects which are understood to have socially constructed meanings rather than intrinsic natures (Mead 1934) in a variety of ways, for example contested political object, commercial entity, sacred object, highly regulated legal object (Franklin and Roberts 2001; Williams *et al.* 2003). How embryos are constituted as social objects is subject to continual and contextualised change.

Casper's (1998a) work on how the fetus is constructed in a variety of ways draws on Mead's (1934) concept of *social objects*, which is extended to include *work objects*, described by Casper as ‘any material entity around which people make meaning and organize their work practices’. This concept helps her to examine how in fetal surgery, ‘constructions of the fetus vary depending upon who cares for it, who is attributing meaning, what the work goals are, and material contingencies such as fetal death’ (Casper 1998a: 19). In addition she draws on the concept of *negotiated order* (Strauss *et al.* 1964, Strauss 1978) in which understandings and more formal rules, norms, definitions and policies are also continually *remade* through ongoing interactions, *i.e.* they are emergent and contingent (Casper 1998b: 381) and usually also relate to work goals. On the concept of negotiated order, Allen (1997) observes that social ordering may not always take the form of face-to-face negotiation, and social order is ‘continuously accomplished rather than negotiated’ (1997: 515). This is important because, as we argue in this paper, emergent meanings and the organisation of particular work practices in relation to embryos may also arise through more subtle processes than overt negotiation. A variety of these meanings can co-exist while achieving sufficient adherence to a broader social order – in this case an official ethical and regulatory formulation – to allow pursuit of an overall work goal. We build on these concepts to explore how practitioners in the contemporary UK ethical framework and regulatory environment relating to reproductive technologies focus on the goals and motives of their work on embryos, and on certain consequences of their work but not others, constituting the embryo in ways that continually ‘remake’ the locally enacted social order. We define our further category of moral work object as a social object around which people make meaning and organise work practices in a morally contested field. The use of the term ‘moral work object’ is not intended to denote a sense that there is a ‘good’ way of constructing the embryo, or that staff simplistically or uncritically adhere to the idea of a ‘normal’ embryo; rather it is used in the more neutral sense of a category of objects around which people make meanings in a morally contested field. We will argue that staff accomplish or ‘remake’ the formally negotiated social order at the same time as constituting the embryo in a variety of forms of ‘moral work object’. More broadly, this paper adds to the small but growing field of sociological studies that explore ethics in clinical settings (de Vries and Conrad 1998, Haimes 2002, Williams 2006).

### Clinical background

The treatment goal of PGD is to produce a ‘healthy baby’. This entails discarding embryos which have not been selected for transfer to the woman's womb for implantation. PGD can be offered to couples who are at risk of having a child with a serious genetic condition, or in some cases, to couples who have experienced repeated miscarriage due to chromosome rearrangements such as reciprocal translocation (Braude *et al.* 2002). IVF is used to create embryos in the laboratory, from which one or two cells, or blastomeres, can be tested for specific genetic disorders. Currently in the UK, up to two unaffected embryos can then be transferred to the woman, where they may successfully implant. PGD embryos affected by the genetic disease being tested for may be donated for research or allowed to perish. PGD is offered in about eight centres in the UK, which must be licensed by the Human Fertilisation and Embryology Authority (HFEA). To date licences have been issued for the testing of about 70 genetic conditions. As well as avoiding termination of an established fetus, one of the key advantages of PGD is to avoid repeated termination of pregnancies following antenatal diagnosis of genetic disease, which may have serious and long-term effects on women/couples (Lavery *et al.* 2002).

In some cases the social and ethical issues pertaining to PGD include or overlap with considerations relating to IVF. As many of the staff we interviewed work across the areas of IVF and PGD, their comments often apply to both fields, but where there are important implications to be drawn from the distinction, we highlight them. The key distinction between PGD and IVF for the purpose of this paper is that PGD embryos may be discarded not only because, as in IVF, they are non-viable or ‘surplus’ to the woman's or couple's goals for family creation, but because they have been diagnosed as being affected by a particular genetic condition that the woman/couple wish to avoid.

## Methods

The project from which this paper reports explored what actual and potential ethical, social, clinical and legal dilemmas genetic developments and new reproductive technologies pose for practitioners, scientists, policy makers and others working in the area of PGD, and the social processes, meanings and institutions that frame and produce ‘ethics’ and ‘ethical problems’. Although there have been a number of studies on the experiences and views of women/couples undergoing PGD and/or IVF (Franklin 1997; Becker 2000), there is a lack of research on the views of staff and policy makers, so this research focused specifically on their experiences and views.

The project used multiple methods to study two sites, both Assisted Conception Units (ACU) in teaching hospitals in England which offer a mixture of National Health Service (NHS), private, or ‘self-funded’ NHS treatment. The clinics provide a range of services including IVF to women and couples who need fertility treatment, and PGD, which requires many of the same procedures and technologies.

Following Ethics Committee approval, our research included observation in clinics; interviews with staff from a range of disciplines including nursing, obstetrics and gynaecology, ultrasonography, embryology, molecular and cyto-genetics, and administration; and ethics discussion groups (EDGs) facilitated by a specialist in medical ethics. This paper draws on the set of 26 staff interviews and five EDGs from our first study site, generated between May and December 2005. Participants were recruited following explanations of the research and informal follow-up approaches from the researchers, and included staff from each of the disciplines working in the clinic. The interviews were conducted as ‘guided conversations’ (Lofland and Lofland 1984), lasting between one and two hours. Open-ended questions and an informal interview schedule were used, with themes including: views on treatment eligibility for PGD; views about the genetic conditions that should be tested for; the status of the embryo; and the value and efficacy of regulatory systems such as the HFEA. Topics for the EDGs were generated from a content analysis of the 26 staff interviews, and by asking participants what issues they felt could usefully be discussed in the groups (Alderson *et al.* 2002). Most of those interviewed participated in an EDG, although a number of staff who were not interviewed also took part. The groups lasted two hours each, and all the discussions were tape-recorded and transcribed.

Transcripts were analysed for this paper using a modified version of the framework approach (Ritchie and Spencer 1994). Sections of the transcripts relating to topics such as the status of the embryo, and allowing embryos to perish, were selected and examined to produce a framework of themes. Sections of the transcripts relating to these themes were then grouped together and analysed further to generate sub-topics. Study numbers are used to protect anonymity, and for the same reason reference to occupations is in general terms rather than specific job titles, so, for example, the category of ‘counsellor’ could include specialist genetics counsellors who provide genetics information or counsellors who work primarily with women/couples on emotional issues. We present quotes selected from the interviews and the EDGs not necessarily to argue for their generalisability but to show the variety of views amongst people doing the same kind of work. We found a similar diversity of views in our second site, but have not included those data in this paper for reasons of space, including the need that would then be generated to explain similarities and differences between the two sites. We wish to stress, therefore, that the views presented here pertain to the medical and wider cultural, historical and legal context in which they occurred. Our findings are therefore limited and we do not make claims as to their generalisability to other sites within the UK or to settings outside the UK.

We were aware of the possible effects that our own role as researchers may have had in generating the focus on the status of the embryo, and possibly creating some ambivalent feelings in staff through asking questions in certain ways and the setting up of the EDGs in a particular format, which provided opportunities for staff to explore potentially challenging topics. Brief telephone-based evaluations of the groups, in which each participant commented on the experience, highlighted the high level of trust generated in the EDGs. A few participants, however, were more constrained than in the interviews, for example, because they did not wish to reveal very personal feelings, including ambivalence, to colleagues. We regard this as support for our view that bringing the two kinds of information together to bear on our discussion is a fruitful approach.

## Themes

In this section of the paper we start with how staff in the interviews regarded the question of the status of the embryo, to illustrate the extent to which the staff held personal views that were in alignment or differed from the current policy formulation, and the ways in which these views linked with particular work goals within IVF and PGD. We then present their views about discarding embryos or making them available for research, and go on to explore some of the strategies they employed for dealing with or reconciling these feelings when they were not in full accord with the ‘official’ account. In the last part of this section we include data from the EDGs to explore some of the ways in which staff negotiate a variety of ethical positions within the institution in pursuit of their overarching work goals.

## Status of the embryo

Many different versions of the status of embryos were produced in the interviews, with some staff holding views that spanned more than one of the categories produced here. Their comments mainly refer to three-to-five-day-old embryos being tested as part of the PGD process. Participants’ views are presented here in terms of the degree to which moral significance is attached to the embryo at different stages.

### A bunch of cells

The first category fits most closely with the official version of the pre-14-day-old embryo as deserving respect but not holding full moral or ‘human’ status. In this category, biological characteristics of embryos were privileged over their social or moral value, for example referring to embryos as ‘material’, ‘just a bunch of cells’, not ‘entitled to any special rights’ (Scientist 3) or ‘just a bundle of cells’ (Embryologist 5). This is the least problematic view because construction of the embryo as ‘material’ means that a relationship with embryos as work objects can be formed that raises the least conflict for staff between one of the effects of IVF and PGD work goals (eventual destruction of embryos) and the embryo's potentially morally contested social value.

### Cells with special potential

In this category, the embryos are still seen as a collection of cells, but as Embryologist 5 says, the embryo is ‘definitely special … because it has this potential’. Similarly, Embryologist 15 sees embryos not as humans but ‘as potential’. Focusing on the potential of the embryo is consistent with the treatment goals of PGD and IVF and, similarly, may raise few ethical problems for staff because the value of embryos is still predominantly in terms of their biological/material value. However, some IVF staff made distinctions about the nature of this potential, linked to differences between the treatment (and work) goals of IVF and PGD. That is, the goal of IVF is to help create a successful pregnancy, while for PGD it is to produce a ‘healthy baby’, which entails the destruction of some embryos that would have been transferred in IVF.

### A life/beings/babies

In contrast to the first two categories, a few participants went a step further and defined the embryo as ‘a life’ (Nurse 4) or ‘the start of life’ (Doctor 24), marking a departure from the previous categories because ‘a life’ seems to signify an increase in social value from the bunch of cells that ‘can't do anything’, or as an entity which is valued for its potential rather than its current status. Further, some staff used the words ‘beings’ and ‘babies’ to describe embryos. This view is usually found in the discourse opposing PGD or research using embryos, so was perhaps more unexpected. Scientist 2 sees embryos as ‘little beings’ from conception, and Doctor 6 tells patients, ‘we’re replacing two embryos or two little babies’. Doctor 14 remembers going to the incubator to ‘say “hello” to my babies’, and says, ‘It is difficult to see them as a cluster of cells because they aren't.’ These participants expressed some mixed feelings when they talked about working with embryos and were more sensitive about the fate of embryos that are not selected. Nurse 4 and Doctor 6 seemed generally more oriented to the work goals of IVF, whereas Doctors 24 and 14, and Scientist 2 were more broadly committed to the PGD goal of helping women/couples to have a ‘healthy baby’.

## Staff perceptions about discarding embryos or donating them for research

Diversity of opinion on the status of the embryo relates in complex ways to views about what can be done with the embryos created. Once again, staff views combined comments about their work practices and goals with their views about the status of embryo.

### Acceptance

As might be expected, those who regarded the embryo as having no special status relied upon these beliefs when explaining why they accepted discarding embryos or donating them for research. Scientist 8 thought it was ‘absolutely fine to discard embryos’ because they have no central nervous system, feeling of pain, consciousness, concept of their own identity, or any interest in their own future existence. Counsellor 17 sees embryos as a ball of cells with no soul, no being, and not in an environment where they’re going to grow and develop into a pregnancy, ‘so research on embryos is something that I have no problem with at all’.

Acceptance of the disposal of PGD embryos when they are ‘affected’, or non-viable is consistent with a construction of the embryo that prioritises the overarching work goal of PGD. Counsellor 10 feels ‘comfortable with the fact that not all embryos will be transferred and that's part of the treatment and that's okay’, and Doctor 24 considers that ‘in a way it is killing … but we are fine balancing it to the bigger social requirements … the end result of why we are doing it, for me is justification by itself’. These views differ from the first form of acceptance because they are not dependent on a view of the embryo as having no special status, and may even coexist with regarding embryos as the start of life, but priority is given to the PGD work goal.

### Potentially problematic views about discarding embryos

Considering the official account of the embryo not having full moral status, the policy formulation accepting research on embryos, and the general acceptance amongst most staff that one of the effects of the treatment goal of PGD is the disposal of more embryos than in IVF, it was interesting to hear expressed potentially problematic feelings about discarding embryos, and not always within what might be thought of as work goal boundaries. Scientist 2, whose work is oriented in the PGD team, referred to ‘throwing away potential people’ and having to decide ‘how to deal with it because I'm actually killing something’, while Nurse 4, whose work did not entail handling embryos, ‘couldn't be an embryologist because … they have to get rid of the embryos that are not used … that would be my problem’. Embryologist 7 feels ‘quite sad’ about disposing of stored embryos: ‘because of death … such a waste of embryos, producing, freezing and discarding them, and treating them as something that's for disposal’; and Embryologist 26 sometimes feels that discarding ‘beautiful’ embryos ‘is actually quite painful’.

Although the variety of feelings reported in these first two themes was clearly only part of our participants’ overall experience, these feelings do represent a more diverse and sometimes ambivalent construction of embryos and are rarely if ever reported. In these comments, we can see a variety of reasons why discarding embryos could be an issue for these members of staff, including the matter of how directly they are involved in this aspect of the overall treatment process (see also Farsides, Williams and Alderson 2005). Thinking about the destruction of embryos as part of the work brings out ambivalent feelings and raises potential problems for some staff with a variety of work goals and points of view on the status of the embryo. The diversity of micro-level work goals, within an overarching work goal of the ACU to offer the option of PGD, may also be constituted by different occupational orientations and religious views, but these did not fall neatly into distinct professional categories.

## Strategies for managing potentially problematic views about embryos

We now turn to some of the strategies staff employ to deal with potentially problematic views about discarding embryos or their donation for research.

### Avoiding thinking about it

Doctor 6 expresses the view that life starts at fertilisation, and says that he tries not to think about embryos being discarded or used for research, but if those embryos are non-viable, he is ‘happy with that’. The concept of ‘viability’ helps him to focus instead on a biologically orientated construction of the embryo and the overarching treatment goal.

### Rationalising/distancing

This leads on to a related strategy of privileging logical or rational arguments over potentially problematic feelings in order to continue the work. Doctor 6 argues that his job ‘has only been brought about through extensive research on normal embryos. It would therefore be very hypocritical of me to then sit here and say I'm unhappy with it, because I mustn't be unhappy, otherwise I wouldn't be here.’ By privileging the logical necessity of embryo research within his IVF work goal of creating life, he achieves less conflict. Embryologist 15 employs a distancing strategy, arguing that thinking of embryos as human beings ‘would drive you crazy. I think it would do your head in if you saw everything as human. I think you don't ever forget the potential of each embryo. But you have to have some sort of distance from them to enable you to work with them, because if you've got any sort of emotional attachment to them … it would be almost impossible to work that way.’ Many of the staff who talked about the embryo as potential tended to focus on the lack of full moral status and therefore did not express strong feelings of ambivalence, but Embryologist 15 describes getting ‘a pang of like, a lost potential’ when embryos do not go on to implantation. These feelings about embryos could make her working life difficult. Constructing embryos as moral work objects in her case means creating some moral and emotional distance from them, so that she can work with them.

### Prioritising reproductive autonomy over personal views

A further position is to prioritise women's/couples’ choice as a work goal over more personal feelings. Embryologist 26 supports abortion and a woman's right to exercise reproductive choice, even if this causes personal conflict: ‘If you feel you've done the best you can for the patient and this is their wishes, it tugs at your heart strings to throw away a beautiful embryo, but if it's what they want, then so be it.’ This stance has similarities to the acceptance of discarding some embryos as part of achieving the goal of PGD to achieve a ‘healthy baby’. In this case, the overall commitment to supporting reproductive autonomy for women is more important than the immediate consequences to some embryos or feelings that may be engendered. The embryo is constructed as a moral work object that may be discarded if required by emphasising women's autonomy within the overall treatment goal.

## Reconciling a diversity of constructions of the embryo as institutionally reflexive work

Strategies for dealing with thoughts and feelings about the embryo may be seen as personally useful to staff in that they contribute to achieving a workable tension between potentially conflicting ethical positions, differences between individual and overarching ACU work goals, and the policy framework for IVF and PGD to which staff are committed. We argue that they also perform institutional kinds of work because they ensure that personal ethical views that are not exactly in accord with the official account are *managed*, and thus may also be seen as a form of institutional reflexivity. Further to the more private forms of managing diversity on behalf of shared work goals, we discerned subtle forms of intra-institutional boundary-work which, reflecting the current national policy, could be seen as attempts to bracket off debate on the status, and destruction, of embryos.

### Normative statements and aligning strategies

Several participants commented in the interviews and EDGs that if one believes that an embryo has full moral status from the moment of conception, then one wouldn't (and possibly shouldn't) be working in PGD or in a unit where destruction of embryos is part of the work.

In EDG 2, Scientist 8 said ‘if any of us believed that human beings were there in those embryos we wouldn't be doing the work we’re doing … I don't think we could possibly do the work with them’, and Doctor 22 and Scientist 29 made similar comments. This expresses the logic of those with views clearly in alignment with the official view of the embryo, but such comments could be perceived by colleagues with a less clearly aligned or more complex view as somewhat prescriptive and silencing, whether or not that effect was intended. Other instances of aligning views around the embryo as moral work object included informal talks between senior and junior staff, such as when Scientist 9 joined the staff and was asked: ‘Do you have any problems working with embryos?’. Doctor 11 recalls: ‘One of our trainees, who ultimately worked in the unit, hadn't thought about that, and was absolutely thrown in the interview process, because she did have feelings, but hadn't had time to think about it’.

In EDG 5, Nurse 35 described how she negotiates between her personal ambivalence and what could be seen as an internalised response to the forms of alignment referred to above: ‘I struggle already with some of the PGD that we do … [then later in the meeting] … But then my argument sometimes is that it's like … if you don't agree with it, you shouldn't be doing, you shouldn't be in that profession … which is why I think it's interesting about what you were saying earlier about how people opt out of doing stuff, because you’re still working for a fertility unit, you’re still working for [institution]. So if [institution] are doing it, whether you’re doing it or not, you still come under that label’. In this case the nurse's personal views and IVF orientation sometimes sat uncomfortably with her identification with the unit's overarching work goals and adherence to the predominant ethical framework, leaving her with ambivalent feelings.

### Prioritising the work goal of being a ‘good’ team

Athough many staff comments reflected the wider ethical acceptance of the destruction of pre-14-day-old embryos, we observed instances in the EDGs of the group accommodating more diverse ethical positions. No direct expressions of belief in the full moral status of embryos were made in the groups, but staff made claims that they still function as a coherent team even when individual views are not always in alignment. In EDG 1, Counsellor 10 said: ‘I think as a PGD team we’re very good and we have meetings on a routine basis … I don't think I've been to a meeting where we haven't discussed one of these issues, and we don't always agree round the table’. This highlights the importance of overarching work goals over individual opinions, including the goal of working well together as a team. This form of collegiate behaviour, with tolerance of diversity as a notable feature, could also include signalling tolerance of what people are not able to say, because it shows respect for people's privacy and perhaps recognition that it is risky to state openly a dissenting or ambivalent view. During EDG 2, when most of the contributions were from those who held the ‘just a ball of cells’ view, Counsellor 28 opened up a space for an alternative view to be expressed: ‘I have no problem discarding embryos, from a personal point of view. But some people do. And I can understand why they do’.

Some of the participants in the EDGs displayed not only tolerance of diverse views but responded to the opportunity to voice their ambivalence with their colleagues. Counsellor 18 spoke about struggling with ethical issues and, in contrast, choosing not to discuss this outside the unit, reflecting on her awareness of public perception of her work: ‘I have, over the years, picked up people's things –‘God, did you read about that?’ You know, that thing, ‘Isn't it disgusting what we do?’ And I'm thinking, you know, actually sometimes I have a real problem justifying what we do, myself. You know, I really do question whether this is right, for whom is it right, why is it right, why is it wrong? And I struggle enough with that without actually other people dealing with that’. In relating the experience of being confronted with other people's ‘disgust’, the counsellor invokes the other participants’ recognition of a common difficulty (‘you know, that thing’) of having one's work disapproved of by the public. This then stands in contrast to her open expression of uncertainty or diversity in the context of the group, and by implication suggests she experiences the team as trustworthy and supportive.

In the third and fourth themes we have explored ways in which staff negotiate with the prevailing social order, *i.e.* the ethical and regulatory formulation that allows for destruction of pre-14-day-old embryos as part of the overall work goals of PGD and IVF. We are building particularly on Casper's (1998a,b) findings because staff working in this morally contested and multidisciplinary field need continually to ‘remake’ aspects of the social order in a similar way to those working in fetal surgery. We argue that staff working in this ACU remake the ethical and regulated social order of work with embryos locally by employing normative, accommodating, and prioritising strategies to achieve shared work goals despite diverse constructions of the embryo and micro-level work goals. Thus the embryo is constructed as a moral work object in diverse forms that allow the staff to do their work.

As Allen (1997) suggests, this points to the value of looking at processes in which social order is ‘continuously accomplished rather than negotiated’ (1997: 515) and in a more subtle way than suggested by Casper. It may be that staff choose to work in the field and particular units because they are in accord with the prevailing ethical framework, and this may be the most comfortable position for some individuals and for particular units as a whole. However, it may produce a self-selected group lacking a diversity of views that could help contribute to a ‘humane’ service (Farsides, Williams and Alderson 2005). Staff whose views are not in close alignment with the predominant ethical framework need to find ways of managing their personal views and feelings, and we argue this may serve both personal and institutional purposes (see also, Hochschild 1984, Fineman 1993). Belonging to a ‘good’ team appears also to be a work goal and this can take priority over some of the uncertainties and diversity expressed. We argue that managing such uncertainties, ambivalence and diversity of micro-level work goals are forms of institutionally reflexive work that further constitute the embryo as ‘moral work object’, because they contribute to the group's ethical and moral consciousness, enhancing the work and the service provided to the unit's clients.

## Discussion and conclusion

In contrast to mass media representations that polarise stances on the ‘embryo question’, we found that staff in one ACU held a variety of beliefs about when life begins, when an embryo becomes morally significant, and the acceptability of discarding embryos or donating them for research. We consider it is worth exploring this variety of views because opening this professional ‘black box’ reveals some potential ambivalence, as suggested by Kerr (2004), which is rarely reported. Further, our findings support Kerr and Cunningham-Burley's (2000) claim that institutional reflexivity may be observed in the setting of boundaries around certain areas for debate and the focus on overarching work goals, rather than some of the consequences, which aids the professional project of expanding ‘ethically acceptable’ clinical genetic services.

We argue further that managing potential diversity and ambivalence when staff find their beliefs do not exactly align with the official policy framework on this issue is a form of negotiating the social order and as such can also be described as institutional work. This involves social practices that have been largely unexplored through policy makers’ attempts to close the debate. Although staff in this unit did not report a requirement to display overtly a ‘correct’ construction in relation to embryos as part of the performance of their work, in more subtle ways they may seek to construct the embryo as a moral work object in forms that ‘fit in’ with the current policy framework and enable them to continue their work with minimal conflict. These attempts may have material consequences, as we saw when Nurse 4 said she could not work in embryology because it would entail direct involvement with disposal of embryos (Farsides, Williams and Alderson 2005).

Focusing on the embryo as ‘potential’, and according ‘respect’ to the embryo, are important concepts in constituting the embryo as a moral work object. In the wider debates on the ‘embryo question’, the word ‘potential’ is given great significance and used, for example by those who take a pro-life stance, to argue that embryos should not be subject to any testing or research that might destroy them. Some of our participants appear to be using the word ‘potential’ in a different, less profound, way, signalling their awareness of this aspect of the ‘specialness’ of embryos, but without according them the same degree of moral significance. This allows concerns about one of the more difficult *consequences* of their work – destruction of embryos – to be managed by some staff so that research and practice can continue. Fundamental questions about the value of human life at the three-day-old embryo stage may be deflected by prioritising instead overarching work goals such as providing women with reproductive choice, helping to create ‘healthy babies’, and being part of a good team.

Our findings support Casper's (1998a) argument that differences between practitioners may be ‘mobilized in certain ways to produce a negotiated order, an outcome that supports the overall institutional goals’ (1998a: 132). However, as Allen (1997) argues, these differences may not necessarily take on a formalised or overt negotiative character as in Casper's study. The negotiation we address is not so much the social order constituted by social relations between particular professional groups; rather, negotiation of the ethical and social order is formalised to a large extent outside, or at a ‘higher level’ bureaucratically speaking, but is then ‘remade’ in the local context. Staff employ strategies for producing a lack of conflict, one of which is to focus on particular work goals to construct the embryo as a moral work object, even when some staff regard the embryo ontologically in ways that could be problematic for them. As a further form of reflexivity, staff may signal tolerance of diversity in subtle ways which minimise overt conflict, thus supporting the work goal of forming a good team.

We argue that our data adds to work on ways in which defining and redefining the biological and moral status of embryos may serve to overcome conflicts and ambivalence and allow for new kinds of projects to develop. Ambivalence can be seen as an expected counter effect to what can arguably be thought of as a particular form of social ordering through reproductive and genetic technologies (avoiding the incidence of serious genetic disorders). Clearly these processes vary over time and take different forms in different places. For example, in contrast to when embryology's future as a discipline entailed ascribing full ‘human’ status at the earliest stage of antenatal development (Morgan, 2003: 271), in the contemporary UK context, the conclusion to be drawn from the policy discourse is that the current ethical formulation is to *defer* full moral status to the embryo in order to allow work on what is regarded as not fully human yet biological ‘material’.

In conclusion, we found a diversity of views, including some ambivalence, amongst staff in one ACU relating to the prevailing policy acceptance of the destruction of pre-14-day-old ‘affected’ or ‘spare’ embryos. Diversity in micro-level work goals did not fall into distinct professional categories although it seems that orientations more in IVF than PGD were associated for some staff with more ambivalence. Subtle distinctions in the staff's micro-level work goals played a significant part in making certain strategies for dealing with potentially problematic views both necessary and successful, at the personal and institutional levels. Some staff engage in the wider project of institutionally reflexive work when they find they need to reconcile aspects of their personal work goals and moral views about embryos with the prevailing ethical and policy framework that supports their clinical and scientific work and allows new projects to develop. In performing this work, staff construct the embryo in a variety of forms of moral work object, allowing attention to shift between micro-level and overarching work *goals*, and away from a more problematic *consequence* of their work, the destruction of human embryos. This adds a further dimension to the concept of moral work object, and adds depth to the understanding of what it takes to maintain the workable tensions, or broader negotiated order, that culminated in the 1990 Act.
